# Diabetes mellitus is associated to high-risk late gadolinium enhancement and worse outcomes in patients with nonischemic dilated cardiomyopathy

**DOI:** 10.1186/s12933-024-02127-z

**Published:** 2024-01-20

**Authors:** Pablo Zulet, Fabián Islas, Marcos Ferrández-Escarabajal, Ana Bustos, Beatriz Cabeza, Sandra Gil-Abizanda, María Vidal, Irene Martín-Lores, Paula Hernández-Mateo, J. Alberto de Agustín, Carmen Olmos

**Affiliations:** 1 Instituto Cardiovascular, Hospital Clínico San Carlos, Instituto de Investigación Sanitaria del Hospital Clínico San Carlos (IdISSC), C/Profesor Martín Lagos s/n, Madrid, 28040 Spain; 2 Servicio de Diagnóstico por la Imagen, Hospital Clínico San Carlos, Madrid, Spain; 3 Universidad Europea de Madrid, Madrid, Spain

**Keywords:** Dilated cardiomyopathy, Diabetes mellitus, Cardiac magnetic resonance imaging, Late gadolinium enhancement

## Abstract

**Background:**

Diabetes mellitus (DM) is associated with a worse prognosis in patients with heart failure. Our aim was to analyze the clinical and imaging features of patients with DM and their association with outcomes in comparison to nondiabetic patients in a cohort of patients with nonischemic dilated cardiomyopathy (DCM).

**Methods:**

This is a prospective cohort study of patients with DCM evaluated in a tertiary care center from 2018 to 2021. Transthoracic echocardiography and cardiac magnetic resonance findings were assessed. A high-risk late gadolinium enhancement (LGE) pattern was defined as epicardial, transmural, or septal plus free-wall. The primary outcome was a composite of heart failure hospitalizations and all-cause mortality. Multivariable analyses were performed to evaluate the impact of DM on outcomes.

**Results:**

We studied 192 patients, of which 51 (26.6%) had DM. The median left ventricular ejection fraction was 30%, and 106 (55.2%) had LGE. No significant differences were found in systolic function parameters between patients with and without DM. E/e values were higher (15 vs. 11.9, *p* = 0.025), and both LGE (68.6% vs. 50.4%; *p* = 0.025) and a high-risk LGE pattern (31.4% vs. 18.5%; *p* = 0.047) were more frequently found in patients with DM. The primary outcome occurred more frequently in diabetic patients (41.2% vs. 23.6%, *p* = 0.017). DM was an independent predictor of outcomes (OR 2.01; *p* = 0.049) and of LGE presence (OR 2.15; *p* = 0.048) in the multivariable analysis. Patients with both DM and LGE had the highest risk of events (HR 3.1; *p* = 0.003).

**Conclusion:**

DM is related to a higher presence of LGE in DCM patients and is an independent predictor of outcomes. Patients with DM and LGE had a threefold risk of events. A multimodality imaging approach allows better risk stratification of these patients and may influence therapeutic options.

**Supplementary Information:**

The online version contains supplementary material available at 10.1186/s12933-024-02127-z.

## Introduction

Diabetes mellitus (DM) may cause cardiac damage due to three main pathways: coronary artery disease, cardiac autonomic neuropathy and cardiomyopathy [1, 2]. Diabetic cardiomyopathy (DbCM) has been defined as the presence of cardiac structural or functional abnormalities in diabetic patients in the absence of significant coronary artery disease, valvular disease, or other conventional cardiovascular risk factors [1, 2].

However, there is currently no universally accepted definition for this entity, and frequently, patients with DM have other concurrent conditions that may contribute to myocardial dysfunction [1].

Imaging features of DbCM comprise left ventricular (LV) hypertrophy, LA dilation, and diastolic dysfunction, but DM can also lead to overt systolic dysfunction [1–5]. The underlying mechanisms that link DM to myocardial damage include upregulation of inflammatory signaling, oxidative stress, renin-angiotensin-aldosterone system hyperactivation, impaired mitochondrial and cardiomyocyte calcium handling, and abnormal myocardial energetics, all of which lead to myocyte dysfunction, apoptosis, fibrosis deposition, and reduced cardiac relaxation and contractility [2]. Other molecular mechanisms considered to play a role in the pathogenesis of DbCM include JunD/peroxisome proliferator-activated receptor-γ (PPAR-γ) overexpression [6] and glycosylation of angiotensin converter enzyme 2 [7].

In any case, DM has been strongly linked with an increased risk of heart failure (HF) hospitalization and mortality and with worse diastolic function parameters in patients with HF, both with preserved and reduced ejection fraction [8–10].

Regarding nonischemic dilated cardiomyopathy (DCM), it has been reported in a few observational studies that patients with DCM and DM have a worse prognosis than nondiabetic patients. This fact has been explained by poorer LV longitudinal and diastolic function and more extensive myocardial fibrosis in diabetic patients [11, 12]. However, despite the increasing interest in this area, previous works have only focused on echocardiographic findings, and data from cardiac magnetic resonance (CMR) imaging are lacking.

Thus, we aimed to describe the potential impact of DM on echocardiographic and CMR features in a large cohort of patients with DCM and its association with outcomes, in comparison to nondiabetic patients.

## Methods

### Study design and ethics

A retrospective cohort study assessing prospectively collected data was conducted at one tertiary care hospital in Spain. The study was approved by the local ethical committee. Informed consent was obtained from all individual participants included in the study.

### Study setting and population

From January 2018 to December 2021, all patients with a diagnosis of DCM evaluated in our tertiary care center were prospectively included in a multipurpose registry.

DCM was defined as the presence of a left ventricular ejection fraction (LVEF) < 45% (including truly DCM with LV dilatation and hypokinetic nondilated cardiomyopathy, as defined by the latest European Society of Cardiology proposal) [13, 14], in the absence of history of myocardial infarction, significant coronary artery disease (defined as > 70% luminal stenosis in a major coronary artery or > 50% in the left main coronary artery, ruled out by invasive angiography or coronary computerized tomography), subendocardial late gadolinium enhancement (LGE), primary valve disease, hypertrophic or arrhythmogenic cardiomyopathy, cardiac amyloidosis, congenital heart disease, and acute myocarditis. Patients with transmural LGE but no history of myocardial infarction and absence of coronary artery disease documented by invasive angiography or coronary computerized tomography (CCT) were included if the LGE distribution was not congruent with an infarct in a specific coronary territory [14].

### Echocardiography and cardiac magnetic resonance imaging

At diagnosis, all patients underwent a transthoracic echocardiogram (Vivid E9: GE Vingmed Ultrasound AS, Horten, Norway) and a CMR with 1.5 Tesla scans (GE Signa HDxT and GE Excite) as part of the diagnostic workup.

LV volumes and systolic and diastolic function were analyzed according to current guidelines [15–17]. Speckle-tracking analysis was performed using dedicated software to evaluate global longitudinal strain (GLS) as an approximation to longitudinal LV function (GE, Echopac PC version 201). Typical four-, two- and three-chamber views were used for this purpose. In the case of atrial fibrillation, the measurement of GLS was obtained as the average of ≥ 3 cardiac cycles.

**Fig. 1:**
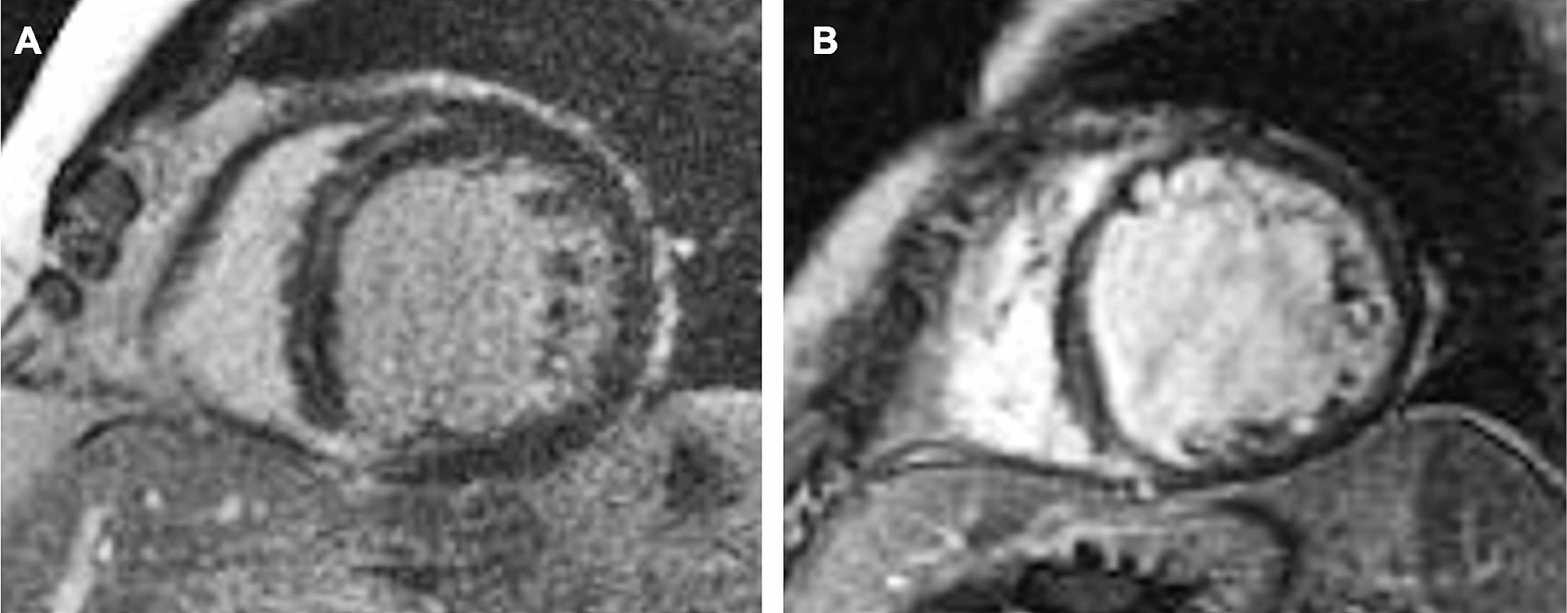
Examples of LGE imaging in two patients with nonischemic dilated cardiomyopathy. Short-axis cardiac magnetic resonance late gadolinium enhancement (LGE) images are shown. Panel **A**: typical septal mid-wall type LGE. Note that a small region of transmural LGE is also present in the inferior septum. Panel **B**: high-risk LGE pattern, with both septal and lateral LGE

CMR included steady-state free precession sequences (SSFP) in 4 chambers, 2 chambers, 3 chambers, and short axes and T1-weighted sequences for late gadolinium enhancement (LGE). LGE imaging was performed 10 to 15 min after the administration of an intravenous bolus of gadolinium-base contrast and was acquired using a phase-sensitive inversion recovery segmented gradient echo sequence. The LGE pattern was assessed visually, and the presence of epicardial, transmural, or septal plus free-wall LGE was identified as high-risk LGE according to previous studies [18, 19]. Images were analyzed with the software Medis Suite, version 3.2 (Medis Medical Imaging Systems. Leiden, The Netherlands). A representative CMR-LGE image of two patients with DCM is shown in Fig. 1.

### Follow-up and outcomes

Patients were followed up at the HF outpatient clinic or general cardiology clinic. Patients with implantable cardiac devices were also regularly followed up in our device clinic every 6 to 12 months, as well as by remote monitoring. Follow-up duration was calculated as the time from DCM diagnosis to the last clinical contact or event occurrence.

Outcome data were obtained from hospital electronic patients records and general practitioners’ records. In addition, mortality was confirmed by the national death register data.

The primary endpoint was a composite of HF hospitalization and all-cause mortality. The secondary endpoints were all-cause mortality, HF hospitalizations, major ventricular arrhythmic (VA) events, and cardiovascular mortality.

Major VA events included appropriate defibrillator therapies (either anti-tachycardia pacing or shocks), sustained monomorphic ventricular tachycardia, sustained polymorphic ventricular tachycardia, resuscitated cardiac arrest, and sudden death during follow-up. Defibrillator therapies were considered as appropriate or inappropriate after careful evaluation by a trained cardiac physiologist.

### Sample size calculation

Assuming a primary event rate of 25%, and up to 4 independent variables, based on the work of Peduzzi et al. [20] the minimum number of cases needed to be included was 160.

### Statistical analysis

Continuous variables are expressed as the mean and standard deviation (SD) or median and interquartile range and were compared with Student’s t test or the Mann‒Whitney U test. Categorical variables were expressed as frequencies and percentages and compared using the chi-square test or Fisher’s exact test. Missing data were < 10% for all variables.

Logistic and Cox proportional hazard regression analyses were performed to identify predictors of outcome. Survival curves were calculated with the Kaplan–Meier method, and cumulative event rates were compared using the log-rank test. Variables considered clinically relevant and those statistically significant in the univariable analyses were included in the multivariable regression analyses. A subset regression procedure was used to select the fittest (lowest Akaike information criterion) and parsimonious multivariable model. 10 outcomes per 1 tested variable was considered to prevent overfitting.

Finally, we performed a logistic regression analysis to determine the variables independently associated with the presence of LGE in our cohort.

All tests were two-sided, and differences were considered statistically significant at *P* values < 0.05. Statistical analysis was performed by using Stata V.16.0 (StataCorp, College Station, Texas, USA).

## Results

### Clinical characteristics and treatment

One hundred and ninety-two patients were included in the study. The mean age of the patients in our cohort was 62.5 (52.5–71.4) years, 66.7% were male, and DM was present in 51 patients (26.6%).

Coronary artery disease was ruled out by invasive coronary angiography in 186 patients (81.2%) and by CCT in the remaining 43 patients.

Epidemiological and clinical characteristics and treatment at the last follow-up are summarized in Table 1. Diabetic patients were significantly older, more frequently male, and had a higher body mass index (*p* < 0.005). Hypertension (HTN), dyslipidemia, chronic obstructive pulmonary disease, atrial fibrillation, and previous stroke, were more frequent in patients with DM, and they had significantly lower estimated glomerular filtration rate (*p* < 0.005). The NYHA functional class was significantly worse in diabetic patients (*p* < 0.005).

**Table 1:** Epidemiological, clinical characteristics and treatment of patients with and without diabetes mellitus

	All patients (*n* = 192)	DM (*n* = 51)	No DM (*n* = 141)	*p* value
Age - years	62.5 [52.5–71.4]	66.6 [56.6–72.3]	59.8 [49-70.8]	**0.020**
Female	64 (33.3)	9 (17.7)	55 (39)	**0.006**
Body mass index	26.4 [23.7–30.9]	28.7 [24.5–33.3]	25.8 [23.1–28.7]	**0.003**
Familial DCM	26 (13.5)	6 (11.7)	20 (14.2)	0.665
Alcohol excess	21 (10.9)	8 (15.7)	13 (9.2)	0.151
Previous chemotherapy	8 (4.2)	1 (2.0)	7 (4.9)	0.454
Left bundle branch block	71 (37.6)	23 (46)	48 (34.5)	0.151
NYHA functional class				
I	81 (42.2)	14 (27.5)	67 (47.5)	**0.035**
II	98 (51.0)	33 (64.7)	65 (46.1)	
III	13 (6.8)	6 (11.8)	7 (5.0)	
Hypertension	86 (44.8)	34 (66.7)	52 (36.9)	**< 0.001**
Dyslipemia	69 (35.9)	33 (64.7)	36 (25.5)	**< 0.001**
Chronic kidney disease	15 (7.9)	7 (14)	8 (5.7)	0.060
Chronic obstructive pulmonary disease	13 (6.8)	7 (13.7)	6 (4.3)	**0.021**
Stroke	16 (8.3)	9 (17.7)	7 (5)	**0.005**
Atrial fibrillation	58 (30.5)	22 (44)	36 (25.7)	**0.016**
**Blood tests**				
NT proBNP (pg/mL)	1135 [388–2837]	1135 [524–2547]	1184.5 [340–2984]	0.943
eGFR (mL/min/1.73 m^2^)	74.2 [59–90]	64.3 [48-80.2]	79.7 [60-90.9]	**0.009**
HbA1c (%)	5.9 [5.6–6.3]	7.2 [6.3–8.1]	5.7 [5.5–6.1]	**< 0.001**
**Medical treatment**				
Loop diuretics	117 (63.6)	33 (67.4)	84 (62.2)	0.523
Beta-blockers	173 (93)	46 (93.9)	127 (92.7)	0.782
Angiotensin-converting enzyme inhibitors/angiotensin II receptor blocker	98 (52.7)	22 (44.9)	76 (55.5)	0.203
Angiotensin-receptor neprilisin inhibitor	74 (39.8)	24 (49)	50 (36.5)	0.125
Mineral receptor antagonists	133 (71.5)	36 (73.5)	97 (70.8)	0.723
Sodium-glucose cotransporter 2 inhibitors	87 (46.3)	32 (62.8)	55 (40.2)	**0.006**
Oral anticoagulants	66 (36.3)	22 (45.8)	44 (32.8)	0.108
**Devices**				
Implantable cardioverter defibrillator	61 (31.8)	22 (43.1)	39 (27.7)	**0.042**
Cardiac resynchronization therapy	32 (16.7)	12 (23.5)	20 (14.2)	0.125

Data are presented as the mean/median [standard deviation/interquartile range] or as frequency (percentage). Values in bold are significant

DM: diabetes mellitus; eGFR: estimated glomerular filtration rate; NT proBNP: N-terminal pro hormone brain natriuretic peptide; NYHA: New York Heart Association

The use of sodium-glucose cotransporter 2 (SGLT2) inhibitors was higher in diabetic patients (62.8% vs. 40.2%; *p* < 0.05), but no significant differences were observed regarding other guideline-recommended HF drugs.

### Echocardiographic and CMR imaging characteristics

A comparison of imaging characteristics in patients with and without DM is shown in Table 2. No significant differences were found in LVEF, LV volumes, or GLS between the two groups. Interestingly, patients with DM had higher E/e values (15 vs. 11.9, *p* = 0.025).

**Table 2:** Imaging findings in patients with and without diabetes mellitus

	All patients (*n* = 192)	DM (*n* = 51)	No DM (*n* = 141)	*p* value
**Transthoracic echocardiogram**				
Left ventricular ejection fraction - %	30 [23.5–38]	31.2 [22.3–36]	30 [24–39]	0.462
LV global longitudinal strain - %	-10 [-12 - -7.1]	-10.1 [-12 - -7.2]	-9.9 [-12 - -7]	0.840
Left atrial volume index (mL/m^2^)	42.8 [33.1–50.7]	42.7 [35.8–47.5]	42.9 [32.4–52.3]	0.782
Moderate or severe mitral regurgitation	59 (34.1)	17 (36.9)	42 (33.1)	0.692
Mechanical dyssynchrony				
Left bundle branch block pattern	24 (24.7)	7 (28)	17 (23.6)	0.661
Apical rocking	42 (32.8)	11 (33.3)	31 (32.6)	0.941
Septal flash	50 (39.1)	17 (51.5)	33 (34.7)	0.089
Diastolic function				
E/A ratio	1.1 [0.7–1.8]	1 [0.6–2.5]	1.1 [0.71–1.7]	0.770
E/e’ ratio	12.7 [9.8–16.4]	15 [11.7–19]	11.9 [8.9–15.8]	**0.025**
Grade I dysfunction	86 (50)	19 (41.3)	67 (53.2)	0.385
Grade II dysfunction	58 (33.7)	18 (39.1)	40 (31.8)	
Grade III dysfunction	28 (16.3)	9 (19.6)	19 (15.1)	
TAPSE (mm)	18 [15–21]	18.5 [15–22]	17 [15–20]	0.083
Right ventricular arterial coupling (mm/mmHg)	0.5 [0.4–0.7]	0.5 [0.4–0.6]	0.6 [0.4–0.7]	0.406
**Cardiac magnetic resonance**				
Left ventricular ejection fraction - %	27 [22–36]	28 [23–35]	26.5 [20–37]	0.736
Right ventricular ejection fraction - %	50 [39–59]	52 [36–60]	49 [39–59]	0.586
Left ventricular mass index (g/m^2^)	99 [72.5–137]	106.5 [86.2–145]	95.6 [67.6-132.9]	0.159
LV end-diastolic volume index (mL/m^2^)	135.5 [111–158]	136.2 [110.4–153]	135.5 [111.5–166]	0.278
LV end-systolic volume index (mL/m^2^)	97 [67.5–124]	93.8 [71–114]	98 [64.6–127]	0.419
Late gadolinium enhancement presence	106 (55.2)	35 (68.6)	71 (50.4)	**0.025**
High risk pattern of late gadolinium enhancement	41 (21.6)	16 (31.4)	25 (18)	**0.047**

Data are presented as the mean [standard deviation] or as frequency (percentage). Values in bold are significant

DM: diabetes mellitus; LV: left ventricle; TAPSE: tricuspid annular plane systolic excursion

LGE was present (LGE+) in 106 (55.2%) patients and was significantly more frequent in patients with DM (68.6% vs. 50.4%; *p* = 0.025). A high-risk LGE pattern was also more frequently observed in the DM group (31.4% vs. 18.5%; *p* = 0.047).

Details about LGE location and distribution are provided in Supplementary Table 1. LGE was more frequently located in the basal segments. Mid-wall septal LGE was found in 63.7% of LGE + patients, and it was the most common pattern. Epicardial LGE was observed in 9.8% of LGE + patients, and in 71% of these cases, mid-wall LGE was also present. Transmural LGE was found in 12.8% of patients.

### Comparison of long-term outcomes of DCM patients with and without DM

**Fig. 2:**
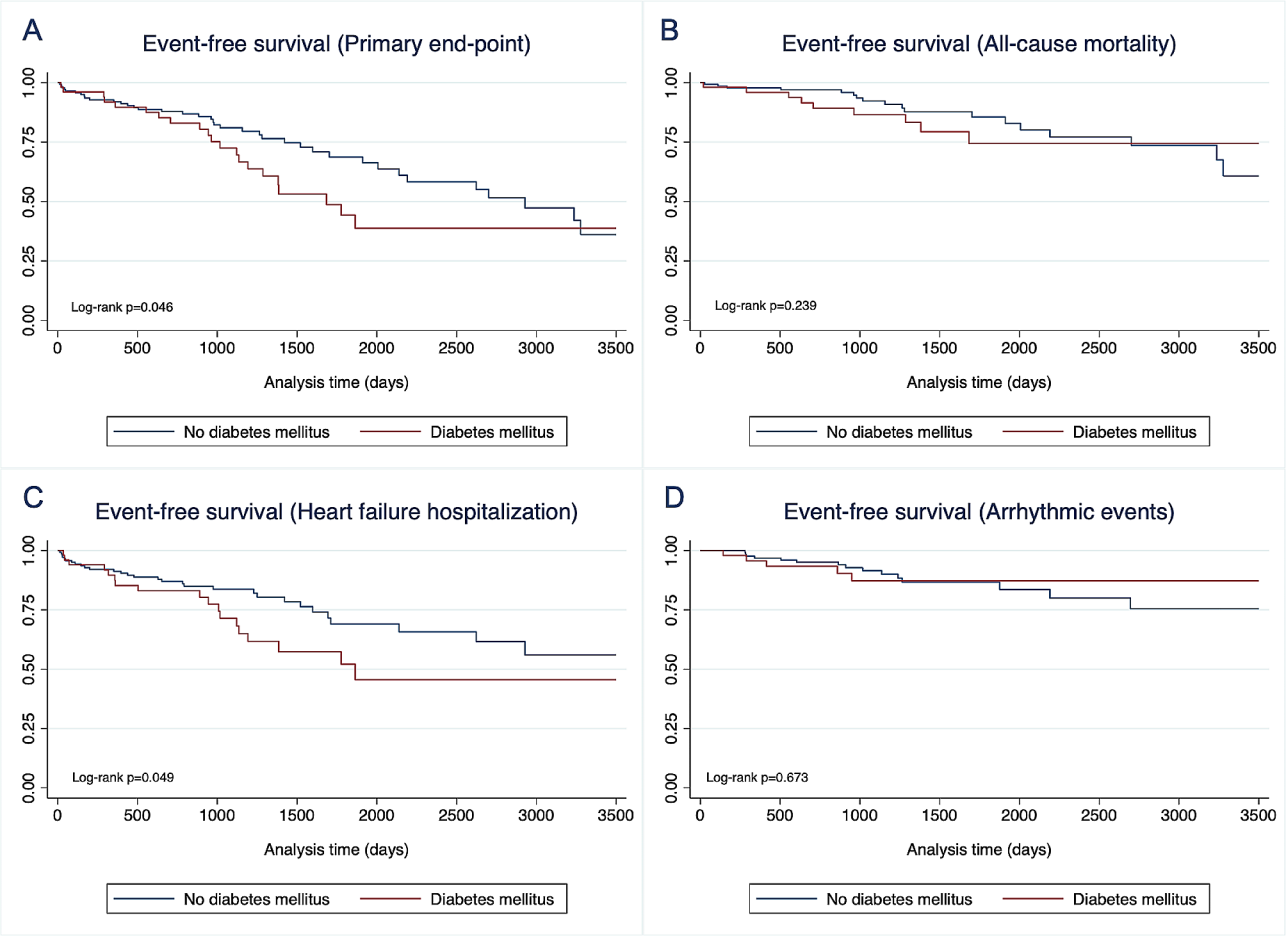
Time-to-event curves according to diabetes mellitus status. Kaplan‒Meier curves according to diabetes mellitus status for survival free from the primary endpoint (Panel **A**), all-cause mortality (Panel **B**), heart failure hospitalization (Panel **C**), and arrhythmic events (Panel **D**)

The median follow-up was 35 (IQR: 21–59) months. Death from any cause or HF hospitalizations occurred more frequently in patients with DM (41.2% vs. 23.6%, *p* = 0.017). No significant differences in VA events were found (Table 3). The survival curves for the primary and secondary outcomes are shown in Fig. 2.

**Table 3:** Events during follow-up in patients with and without diabetes mellitus

	All patients (*n* = 192)	DM (*n* = 51)	No DM (*n* = 141)	*p* value
Heart failure hospitalization or all-cause mortality	54 (28.3)	21 (41.2)	33 (23.6)	**0.017**
All-cause mortality	30 (15.6)	12 (23.5)	18 (12.8)	0.070
Heart failure hospitalization	51 (26.7)	20 (39.2)	31 (22.1)	**0.018**
Cardiovascular mortality	14 (7.3)	5 (9.8)	9 (6.4)	0.421
Ventricular arrhythmic events	24 (12.5)	7 (13.7)	17 (12.1)	0.757

Data are presented as frequencies (percentages). Values in bold are significant

DM: diabetes mellitus

### Association of DM with LGE in patients with DCM and its impact on outcomes

Univariable and multivariable logistic regression analyses were performed to find variables associated with the presence of LGE and a high-risk LGE pattern (Supplementary Tables 2 and 3).

DM and male sex were the only variables independently associated with LGE, whereas DM and age were for the detection of a high-risk LGE pattern (Table 4).

**Table 4:** Multivariable regression analysis for the presence of late gadolinium enhancement and a high-risk pattern

**LATE GADOLINIUM ENHANCEMENT**			
	**Odds ratio**	**95% confidence interval**	***P*** **value**
Diabetes mellitus	2.15	1.05–4.81	**0.048**
Male	2.69	1.34–5.41	**0.005**
**HIGH-RISK LATE GADOLINIUM ENHANCEMENT**			
	**Odds ratio**	**95% confidence interval**	***P*** **value**
Diabetes mellitus	3.24	1.40–7.50	**0.006**
Age (for each year)	0.97	0.94–0.99	**0.027**

Values in bold are significant

### Association of DM and LGE with long-term outcomes in DCM patients

To further evaluate the relationship between DM, LGE, and outcomes, multivariable logistic and Cox proportional hazard regression analyses were performed (Supplementary Table 4).

We included in the models the variables that were considered relevant and statistically significant in the univariable analysis for the primary outcome of death from any cause or HF hospitalization: male sex, DM, HTN, LVEF, LV systolic volume index, and LGE presence, as well as the interaction between DM and LGE.

Variables independently associated with the primary outcome in the multivariable logistic regression analyses were LVEF, male sex, and DM. In the Cox regression analysis, variables independently associated with the occurrence of the primary outcome during follow-up were LVEF, male sex, and the interaction between DM and LGE presence (Table 5).

**Table 5:** Multivariable regression analysis for the occurrence of the primary endpoint defined as all-cause mortality or heart failure hospitalization

**LOGISTIC REGRESSION ANALYSIS**			
	**Odds ratio**	**95% confidence interval**	***P*** **value**
Left ventricular ejection fraction	0.94	0.91–0.98	0.002
Male	1.88	0.95–4.02	0.071
Diabetes mellitus	2.01	1.01–4.03	0.049
**COX PROPORTIONAL HAZARD ANALYSIS**			
	**Hazard ratio**	**95% confidence interval**	***P*** **value**
Left ventricular ejection fraction	0.97	0.94–0.99	0.033
Male	1.80	0.99–3.26	0.053
Interaction between diabetes mellitus and late gadolinium enhancement	2.12	1.18–3.81	0.012

**Fig. 3:**
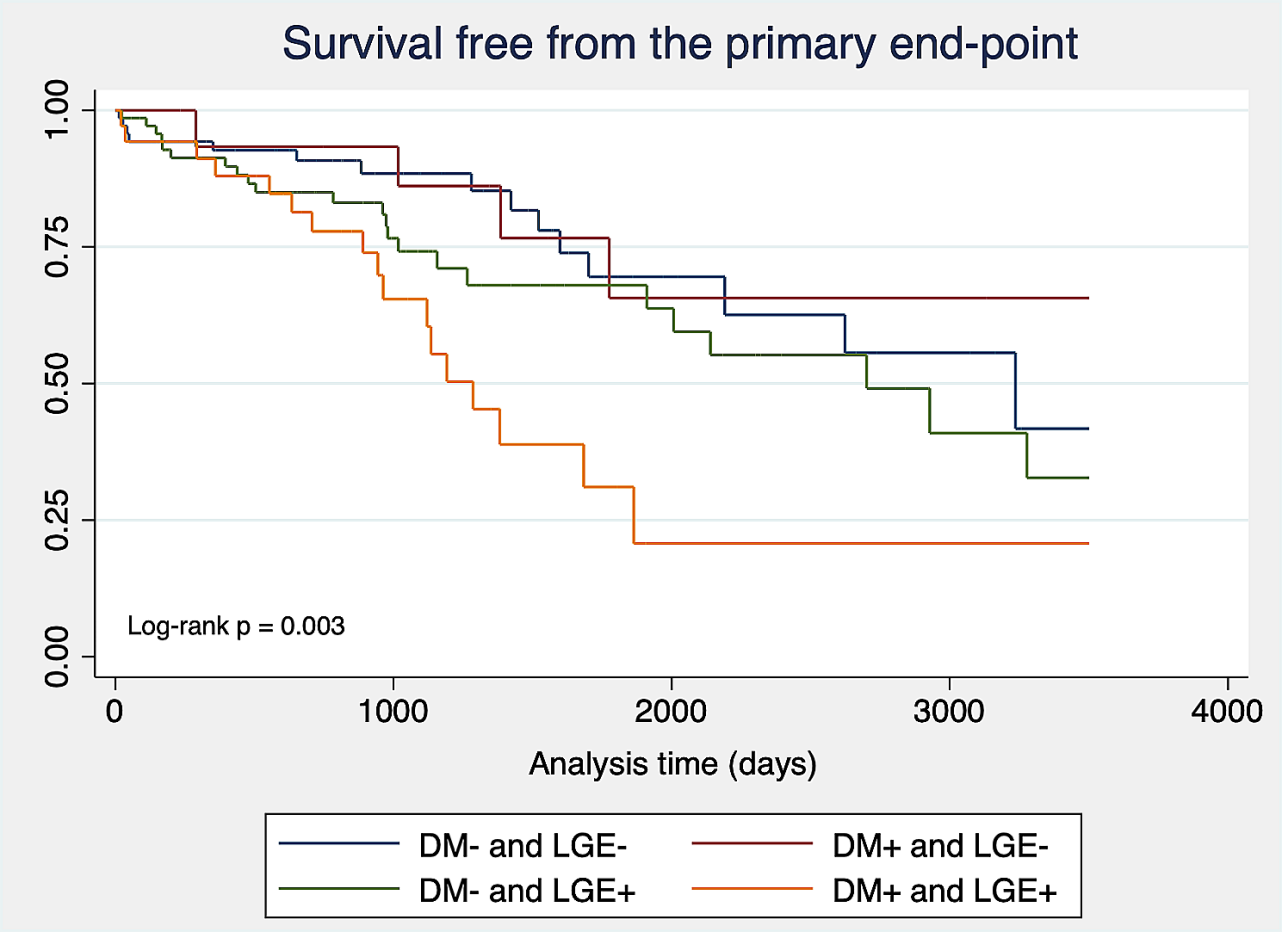
Survival free from the primary endpoint by diabetes mellitus and LGE status. Kaplan‒Meier curves for survival free from the primary endpoint according to diabetes mellitus (DM) and late gadolinium enhancement (LGE) status. Patients with DM and LGE + had the highest incidence of the primary endpoint

Finally, the cohort was divided into 4 groups depending on the presence of DM and LGE. Patients with DM and the absence of LGE (HR 0.94 [95% CI 0.38–2.34]) had a similar risk of events as nondiabetic patients with LGE+ (HR 1.37 [95% CI 0.72–2.61]), whereas those with DM and LGE + had the highest incidence of the primary endpoint (HR 3.10, [95% CI 1.56–6.14]; *p* = 0.003) (Fig. 3).

## Discussion

This study has examined the impact of DM on cardiac structure and function in patients with nonischemic DCM using both echocardiography and CMR, as well as its association with outcomes. There were several main findings. First, DM represented 1 out of 4 patients with DCM, and they had worse diastolic function parameters despite no differences found in LVEF, LV volumes, and GLS in comparison to nondiabetics. Second, DM was strongly associated with the presence of both LGE and LGE high-risk pattern. Third, DM-DCM patients had a worse prognosis, with a higher cumulative incidence of the composite endpoint of HF hospitalization and all-cause mortality than patients without DM. Finally, the association of DM and LGE seems to be a hallmark of particularly poor outcomes.

Very few studies have focused on the potential impact of DM in the specific setting of DCM. Tanaka et al. evaluated with echocardiography 206 patients with DCM, and found that diabetics had significantly lower GLS than non-diabetics, despite similar LVEF [12].

Sakakibara et al. studied 102 patients with DCM who underwent echocardiography and cardiac catheterization with endomyocardial biopsy, of which 30 had DM [11]. No significant differences were found regarding LVEF, LV volumes, or LV dP/dt between patients with and without DM. However, specimens from diabetic patients had a disorganized mitochondrial arrangement and higher interstitial collagen accumulation than those from nondiabetics.

Similar to these findings, we did not find significant differences in LVEF or LV volumes between patients with and without DM. Notably, and in line with their experience, patients with DM in our cohort had worse diastolic function.

Concerning CMR features and, particularly, LGE, the vast majority of studies in patients with DM have concentrated on ischemic heart disease [21, 22], and very little is known regarding nonischemic LGE implications. Pua et al. recently analyzed the impact of DM on myocardial fibrosis in asymptomatic individuals with HTN, and found that diabetics had lower strain and higher replacement fibrosis, as well as upregulation of GDF-15 (growth differentiation factor 15), which was independently associated with replacement myocardial fibrosis, suggesting that inflammatory and immune mechanics could mediate fibrosis in this population [23].

Additionally, a cross-sectional study, that included DM individuals who underwent stress-perfusion CMR, found that nonischemic LGE was present in 9.5% of patients, and they had higher E/e values, LV mass, and left atrial volume index [24]. More recently, Zhang et al. evaluated 235 patients with ischemic (48%) and nonischemic DCM and observed that patients with DM (*n* = 158) had lower global longitudinal strain by CMR and, more frequently, LGE (72.2% vs. 32.5%). However, ischemic cardiomyopathy was 3 times more frequent in the group of patients with DM, which might have influenced findings regarding LGE. Another main shortcoming when interpreting their results is that the LGE pattern (ischemic vs. no ischemic) was not described [25].

To the best of our knowledge, the only dedicated study that analyzed CMR findings in patients with DM and DCM was carried out by Shen et al. The authors studied 435 patients with DCM who underwent CMR, of which 93 had DM. In comparison to nondiabetics, those with DM had higher mass and lower radial and longitudinal strain, despite no differences in LVEF. Interestingly, contrary to what has been previously found in other clinical settings, patients with DM had no differences in LGE burden (70 vs. 66%) compared to nondiabetics. Due to the cross-sectional design, the clinical impact of DM on long-term outcomes was lacking [26].

Thus, in the present study, we describe for the first time specific CMR features of DM patients with DCM and their association with prognosis. We have observed a higher proportion of LGE presence, and, more importantly, of a high-risk LGE pattern, in DM-DCM patients, compared to nondiabetics. In addition, DM was independently associated with the presence of both LGE and high-risk LGE in our cohort.

Recently, three different phenotypes of DCM have been proposed: mild nonfibrotic, biventricular systolic dysfunction, and profibrotic metabolic [27]. The latter was associated with the highest rates of DM and mid-wall myocardial fibrosis was universal. Data from our cohort support the assumption that DM could lead to the further development of myocardial fibrosis in patients with DCM.

Our results also highlight that DM is strongly associated with an increased risk of events, particularly for HF hospitalization, in line with previous works [11, 12]. The multivariable analysis has revealed that the only independent imaging predictors for HF hospitalization and death in our population were LVEF and LGE presence, which had a significant interaction with DM status. LVEF is a well-known marker of poor outcomes, and several studies have demonstrated that LGE is associated with an increased risk of death, particularly from VA events [28, 29], in patients with DCM. The extent of LGE and the distribution are also main predictors of events [18, 19].

A previous study analyzing patients with suspicion of ischemic heart disease who underwent dobutamine stress CMR also found a strong interaction between DM and LGE. The combination of DM and LGE presence was associated with a twofold higher risk of mortality compared to LGE positive patients without DM [22]. Remarkably, in our cohort, which evaluates a completely different clinical scenario, patients with both LGE and DM also had the highest (three-time higher) risk for events.

In the last few years, SGLT2 inhibitors have been associated with reverse cardiac remodeling and reduction in interstitial myocardial fibrosis in preclinical studies [30]. Future studies are needed to evaluate its impact on LGE evolution in DCM.

### Study limitations

Our study has some limitations. First, this is a single-center, observational study with both the inherent limitations of this design.

Second, tissue characterization data using CMR, including T1 and T2 mapping and extracellular volume, were not included since these sequences were performed in a small proportion of the cohort. Parametric techniques could open up further insights into the impact of DM on cardiac structure and function and may provide incremental prognostic information in future studies. Third, the impact of genetic variants on myocardial fibrosis extension and clinical outcomes in patients with DCM is well documented. However, as only 64 patients (27.9% of the population of our cohort) had genetic testing information available, its potential influence was not assessed in the current analyses.

Fourth, the inclusion of anti-tachycardia pacing and shocks as major VA events may have led to an overestimation of this secondary endpoint, as some VA episodes treated by the device could have been self-limited.

Finally, medical treatment was not included in the prognostic models. Several reasons were taken into account: medications were registered close to the time of diagnosis and are subject to change over time, and their use is influenced by factors other than the disease itself, including patient tolerance, clinician and patient preferences, and renal function. In the case of SGLT2 inhibitors, dedicated clinical trials for HF were not yet published when our registry began.

We also did not evaluate the use and influence on outcomes of specific antidiabetic treatment or anti-arrhythmic drugs in our population.

## Conclusions

DM confers a high-risk profile to DCM patients and is associated with a higher presence of LGE and adverse events. The association of DM and LGE was a marker of particularly poor outcomes. A multimodality imaging approach allows better risk stratification of these patients and may influence therapeutic management.

### Electronic supplementary material

Below is the link to the electronic supplementary material.

**Supplementary Table 1**. LGE patterns in patients with and without diabetes mellitus. **Supplementary Table 2**. Univariable regression analysis for the presence of late gadolinium enhancement. **Supplementary Table 3**. Univariable regression analysis for the presence of a high-risk late gadolinium enhancement pattern. **Supplementary Table 4**. Logistic and Cox univariable regression analysis for the occurrence of the primary end-point defined as all-cause mortality or heart failure hospitalization.

## Data Availability

The datasets used and/or analysed during the current study are available from the corresponding author on reasonable request.

## Abbreviations

CCT: Coronary computerized tomography
CI: Confidence interval
CMR: Cardiac magnetic resonance
DCM: Nonischemic dilated cardiomyopathy
DbCM: Diabetic cardiomyopathy
DM: Diabetes mellitus
GLS: Global longitudinal strain
HF: Heart failure
HR: Hazard ratio
IQR: Interquartile range
LA: Left atrium
LAVI: Left atrium volume index
LGE: Late gadolinium enhancement
LV: Left ventricle
LVEF: Left ventricular ejection fraction
SD: Standard deviation
SGLT2: Sodium-glucose cotransporter 2
VA: Ventricular arrhythmia
