# Supplementary material for: Diabetes mellitus is associated to high-risk late gadolinium enhancement and worse outcomes in patients with nonischemic dilated cardiomyopathy
Source: Cardiovasc Diabetol. 2024 Jan 20;23:35. doi: 10.1186/s12933-024-02127-z (PMC10800059; doi:10.1186/s12933-024-02127-z)
Supplement: Supplementary file 1 — Supplementary Table 1. LGE patterns in patients with and without diabetes mellitus. Supplementary Table 2. Univariable regression analysis for the presence of late gadolinium enhancement. Supplementary Table 3. Univariable regression analysis for the presence of a high-risk late gadolinium enhancement pattern. Supplementary Table 4. Logistic and Cox univariable regression analysis for the occurrence of the primary end-point defined as all-cause mortality or heart failure hospitalization. [file 12933_2024_2127_MOESM1_ESM.docx]

Supplementary Table 1. LGE patterns in patients with and without diabetes mellitus

| **LGE distribution in LGE+ patients** | **Total (n=106)** | **DM**  **(n=35)** | **No DM**  **(n=71)** |
| --- | --- | --- | --- |
| Mid-wall | 68 (63.7) | 23 (64.7) | 45 (63.2) |
| Epicardial | 10 (9.8) | 2 (5.9) | 8 (11.8) |
| Epicardial + mid-wall | 14 (12.8) | 4 (11.8) | 9 (13.2) |
| Transmural | 14 (12.8) | 6 (17.7) | 7 (10.3) |
|  |  |  |  |
| Basal segments | 81 (76.5) | 26 (73.5) | 55 (77.9) |
| Mid | 54 (51) | 19 (52.9) | 35 (49.8) |
| Apical | 19 (17.6) | 8 (23.5) | 10 (14.7) |
|  |  |  |  |
| Septal | 31 (29.4) | 11 (32.4) | 20 (27.9) |
| Lateral | 14 (12.8) | 5 (14.7) | 8 (11.8) |
| Inferior | 14 (12.8) | 5 (14.7) | 8 (11.8) |
| Anterior | 2 (2) | 0 (0) | 2 (3) |
| Septal + lateral | 25 (24.6) | 9 (26.5) | 17 (23.6) |
| RV insertion points | 20 (18.4) | 4 (11.7) | 16 (21.9) |

Data are presented as frequency (percentage).

DM: diabetes mellitus; LGE: late gadolinium enhancement; RV: right ventricle

Supplementary Table 2. Univariable regression analysis for the presence of late gadolinium enhancement.

| **Variables** | **Odd ratio** | **95% confidence interval** | **P value** |
| --- | --- | --- | --- |
| **Clinical variables** |  |  |  |
| Age | 1 | 0.98-1.02 | 0.822 |
| Female | 0.30 | 0.16-0.57 | **0.000** |
| Familial DCM | 3.98 | 0.83-19.06 | 0.084 |
| Alcohol excess | 1.59 | 0.6-4.27 | 0.354 |
| Previous chemotherapy | 0.8 | 0.19-3.36 | 0.757 |
| Left bundle branch block | 0.57 | 0.32-1.04 | 0.068 |
| Diabetes mellitus | 2.16 | 1.1-4.25 | **0.026** |
| Hypertension | 1.05 | 0.59-1.85 | 0.879 |
| Dyslipemia | 1.91 | 1.04-3.50 | **0.038** |
| Chronic kidney disease | 2.4 | 0.74-7.82 | 0.147 |
| Chronic obstructive pulmonary disease | 1.32 | 0.42-4.20 | 0.635 |
| Stroke | 2.62 | 0.81-8.43 | 0.107 |
| Atrial fibrillation | 1.22 | 0.65-2.27 | 0.537 |
| **Blood tests** |  |  |  |
| NT proBNP | 1.00 | 0.99-1.00 | 0.328 |
| eGFR | 1.01 | 0.99-1.02 | 0.388 |
| HbA1c | 1.34 | 0.99-1.81 | 0.052 |
| **Imaging features** |  |  |  |
| Left ventricular ejection fraction | 1 | 0.97-1.03 | 0.878 |
| LV end-diastolic volume (index) | 1 | 0.99-1.01 | 0.715 |
| LV global longitudinal strain | 1 | 0.92-1.08 | 0.933 |
| Apical rocking | 0.40 | 0.19-0.85 | **0.018** |
| Moderate or severe mitral regurgitation | 1.03 | 0.55-1.93 | 0.933 |
| Moderate or severe tricuspid regurgitation | 0.87 | 0.33-2.25 | 0.767 |
| TAPSE | 0.92 | 0.85-0.99 | **0.029** |
| Right ventricular arterial coupling | 0.55 | 0.16-1.87 | 0.337 |
| Right ventricular ejection fraction | 0.99 | 0.97-1.01 | 0.171 |
| E/e’ ratio | 0.99 | 0.94-1.04 | 0.679 |
| Left atrial volume index | 1 | 0.99-1.02 | 0.673 |

Values in bold are significant.

eGFR: estimated glomerular filtration rate; DCM: dilated cardiomyopathy; NT proBNP: N-terminal pro hormone brain natriuretic peptide; LV: left ventricle; TAPSE: tricuspid annular plane systolic excursion

Supplementary Table 3. Univariable regression analysis for the presence of a high-risk late gadolinium enhancement pattern.

| **Variables** | **Odd ratio** | **95% confidence interval** | **P value** |
| --- | --- | --- | --- |
| **Clinical variables** |  |  |  |
| Age | 0.98 | 0.95-1 | 0.052 |
| Female sex | 0.57 | 0.26 | 0.158 |
| Familial DCM | 4.58 | 1.33-15.75 | **0.016** |
| Alcohol excess | 1.08 | 0.33-3.57 | 0.903 |
| Previous chemotherapy | 2.75 | 0.60-12.5 | 0.191 |
| Left bundle branch block | 0.81 | 0.39-1.67 | 0.569 |
| Diabetes mellitus | 2.08 | 1-4.34 | **0.049** |
| Hypertension | 0.65 | 0.32-1.33 | 0.238 |
| Dyslipemia | 1.76 | 0.87-3.56 | 0.114 |
| Chronic kidney disease | 1.99 | 0.64-6.18 | 0.236 |
| Chronic obstructive pulmonary disease | 1.09 | 0.29-4.19 | 0.892 |
| Stroke | 1.74 | 0.57-5.33 | 0.331 |
| Atrial fibrillation | 1.47 | 0.70-3.04 | 0.306 |
| **Blood tests** |  |  |  |
| NT proBNP | 1.00 | 0.99-1.00 | 0.276 |
| eGFR | 0.99 | 0.98-1.01 | 0.693 |
| HbA1c | 1.26 | 0.96-1.64 | 0.092 |
| **Imaging features** |  |  |  |
| Left ventricular ejection fraction | 1.01 | 0.98-1.05 | 0.450 |
| LV end-diastolic volume (index) | 1 | 0.99-1.01 | 0.935 |
| LV global longitudinal strain | 0.92 | 0.83-1.03 | 0.132 |
| Apical rocking | 0.35 | 0.13-1.02 | 0.055 |
| Moderate or severe mitral regurgitation | 1.25 | 0.58-2.65 | 0.570 |
| Moderate or severe tricuspid regurgitation | 0.39 | 0.09-1.78 | 0.222 |
| TAPSE | 0.99 | 0.91-1.08 | 0.897 |
| Right ventricular arterial coupling | 0.73 | 0.13-4.05 | 0.717 |
| Right ventricular ejection fraction | 1.01 | 0.99-1.04 | 0.234 |
| E/e’ ratio | 0.98 | 0.92-1.04 | 0.557 |
| Left atrial volume index | 0.99 | 0.97-1.02 | 0.582 |

Values in bold are significant.

eGFR: estimated glomerular filtration rate; DCM: dilated cardiomyopathy; NT proBNP: N-terminal pro hormone brain natriuretic peptide; LV: left ventricle; TAPSE: tricuspid annular plane systolic excursion

Supplementary Table 4. Logistic and Cox univariable regression analysis for the occurrence of the primary end-point defined as all-cause mortality or heart failure hospitalization.

| **Variables** | **Odd ratio** | **95% CI** | **P value** | **Hazard ratio** | **95% CI** | **P value** |
| --- | --- | --- | --- | --- | --- | --- |
| Age | 1.01 | 0.99-1.03 | 0.290 | 1.01 | 0.99-1.03 | 0.286 |
| Female | 0.49 | 0.25-0.96 | **0.038** | 0.47 | 0.27-0.85 | **0.012** |
| Familial DCM | 1.21 | 0.34-4.31 | 0.34 | 0.68 | 0.23-1.95 | 0.470 |
| Alcohol excess | 3.63 | 0.135-9.74 | **0.011** | 2.86 | 1.44-5.68 | **0.003** |
| Previous chemotherapy | 1.45 | 0.33-6.45 | 0.624 | 1.14 | 0.34-3.77 | 0.832 |
| Left bundle branch block | 1.59 | 0.85-2.96 | 0.144 | 0.87 | 0.52-1.46 | 0.593 |
| Diabetes mellitus | 2.49 | 1.28-4.83 | **0.007** | 1.61 | 0.97-2.67 | 0.065 |
| Hypertension | 1.63 | 0.89-2.99 | 0.111 | 1.55 | 0.95-2.53 | 0.083 |
| Dislipemia | 2.43 | 1.31-4.52 | **0.005** | 1.79 | 1.10-2.93 | **0.020** |
| Chronic kidney disease | 1.81 | 0.63-5.24 | 0.273 | 1.88 | 0.84-4.19 | 0.123 |
| Chronic obstructive pulmonary disease | 2.48 | 0.80-7.70 | 0.117 | 3.17 | 1.40-7.18 | **0.006** |
| Stroke | 3.73 | 1.29-10.80 | **0.015** | 1.43 | 0.71-2.86 | 0.313 |
| Atrial fibrillation | 1.44 | 0.76-2.74 | 0.264 | 1.85 | 1.10-3.11 | **0.021** |
| **Blood tests** |  |  |  |  |  |  |
| NT proBNP | 1.00 | 0.99-1.00 | 0.264 | 1.00 | 1.00-1.01 | **0.001** |
| eGFR | 0.98 | 0.96-0.99 | **0.005** | 0.99 | 0.98-1.00 | 0.079 |
| HbA1c | 1.31 | 1.01-1.71 | **0.043** | 1.08 | 0.93-1.27 | 0.293 |
| **Imaging features** |  |  |  |  |  |  |
| Left ventricular ejection fraction | 0.94 | 0.91-0.98 | **0.002** | 0.97 | 0.94-0.99 | **0.018** |
| LV end-diastolic volume (index) | 1.01 | 1-1.02 | 0.073 | 1 | 1-1.01 | 0.258 |
| LV global longitudinal strain | 1.02 | 0.94-1.11 | 0.677 | 1.08 | 1.01-1.15 | **0.033** |
| Apical rocking | 0.47 | 0.21-1.06 | 0.067 | 0.48 | 0.23-0.99 | **0.046** |
| Moderate or severe mitral regurgitation | 1.59 | 0.5-5.04 | 0.433 | 1.9 | 0.70-5.11 | 0.206 |
| Moderate or severe tricuspid regurgitation | 2.28 | 0.42-12.39 | 0.341 | 2.3 | 0.66-7.96 | 0.189 |
| TAPSE | 0.97 | 0.9-1.05 | 0.433 | 0.91 | 0.86-0.97 | **0.004** |
| Right ventricular arterial coupling | 0.55 | 0.14-2.08 | 0.375 | 0.22 | 0.07-0.71 | **0.012** |
| Right ventricular ejection fraction | 0.98 | 0.96-1 | **0.04** | 0.97 | 0.96-0.99 | **0.001** |
| E/e’ ratio | 1.04 | 0.99-1.1 | 0.101 | 1.04 | 1-1.07 | **0.047** |
| Left atrial volume index | 1.01 | 1-1.03 | 0.104 | 1.01 | 1-1.02 | 0.243 |
| Late gadolinium enhancement presence | 1.75 | 0.95-3.25 | 0.074 | 1.83 | 1.09-3.07 | **0.021** |
| High risk pattern of late gadolinium enhancement | 1.38 | 0.67-2.82 | 0.383 | 1.11 | 0.62-1.96 | 0.732 |

Values in bold are significant. CI: confidence intervals.

eGFR: estimated glomerular filtration rate; DCM: dilated cardiomyopathy; NT proBNP: N-terminal pro hormone brain natriuretic peptide; LV: left ventricle; TAPSE: tricuspid annular plane systolic excursion
